# Decreased functional connectivity within a language subnetwork in benign epilepsy with centrotemporal spikes

**DOI:** 10.1002/epi4.12051

**Published:** 2017-04-27

**Authors:** Colm J. McGinnity, Anna B. Smith, Siti N. Yaakub, Sofia Weidenbach Gerbase, Anya Gammerman, Adam L. Tyson, Tiffany K. Bell, Marwa Elmasri, Gareth J. Barker, Mark P. Richardson, Deb K. Pal

**Affiliations:** ^1^ Department of Basic and Clinical Neuroscience Institute of Psychiatry, Psychology & Neuroscience King's College London London United Kingdom; ^2^ Faculty of Life Sciences & Medicine School of Bioscience Education King's College London London United Kingdom; ^3^ Department of Forensic and Neurodevelopmental Sciences Institute of Psychiatry, Psychology & Neuroscience King's College London London United Kingdom; ^4^ Centre for Developmental Neurobiology Institute of Psychiatry, Psychology & Neuroscience King's College London London United Kingdom; ^5^ Department of Neuroimaging Institute of Psychiatry, Psychology & Neuroscience King's College London London United Kingdom

**Keywords:** BECTS, fMRI, Literacy, Language, Rolandic epilepsy

## Abstract

**Objective:**

Benign epilepsy with centrotemporal spikes (BECTS, also known as Rolandic epilepsy) is a common epilepsy syndrome that is associated with literacy and language impairments. The neural mechanisms of the syndrome are not known. The primary objective of this study was to test the hypothesis that functional connectivity within the language network is decreased in children with BECTS. We also tested the hypothesis that siblings of children with BECTS have similar abnormalities.

**Methods:**

Echo planar magnetic resonance (MR) imaging data were acquired from 25 children with BECTS, 12 siblings, and 20 healthy controls, at rest. After preprocessing with particular attention to intrascan motion, the mean signal was extracted from each of 90 regions of interest. Sparse, undirected graphs were constructed from adjacency matrices consisting of Spearman's rank correlation coefficients. Global and nodal graph metrics and subnetwork and pairwise connectivity were compared between groups.

**Results:**

There were no significant differences in graph metrics between groups. Children with BECTS had decreased functional connectivity relative to controls within a four‐node subnetwork, which consisted of the left inferior frontal gyrus, the left superior frontal gyrus, the left supramarginal gyrus, and the right inferior parietal lobe (p = 0.04). A similar but nonsignificant decrease was also observed for the siblings. The BECTS groups had significant increases in connectivity within a five‐node, five‐edge frontal subnetwork.

**Significance:**

The results provide further evidence of decreased functional connectivity between key mediators of speech processing, language, and reading in children with BECTS. We hypothesize that these decreases reflect delayed lateralization of the language network and contribute to specific cognitive impairments.

**Key Points**

- Cognitive impairments such as reading disability and speech sound disorder are recognized in both children with BECTS and their siblings
- The BECTS group had decreased functional connectivity relative to controls within key constituents of the language network
- A similar decrease was observed for siblings, but this did not reach statistical significance
- Children with BECTS also had increased connectivity within a frontal subnetwork

Benign childhood epilepsy with centrotemporal spikes (BECTS), or Rolandic epilepsy (RE), is an idiopathic localization‐related (i.e., focal) electroclinical syndrome that has an annual incidence of approximately 21 per 100,000 in children younger than 15 years of age and constitutes approximately 8–25% of all childhood epilepsies.^1^ The classic sensorimotor seizures, which affect the lower face, mouth, and vocal tract, are infrequent and typically remit during teenage years. Patients are often managed without pharmacotherapy.^2^

Behavioral and cognitive impairments are increasingly recognized in BECTS. For example, we found that 67% of children with BECTS have attention impairments, 54% have language impairments, and 42% have reading disability;^3^, ^4^, ^5^ our meta‐analyses identified moderate effect sizes for reading and language impairments—Cohen's d = 0.7.^3^ Speech dyspraxia^6^ is common.

A causal genetic variant in the *PAX6* gene has been identified for centrotemporal spikes;^7^ and a susceptibility locus for speech sound disorder has been identified,^6^ as well as for associated impairments.^8^, ^9^ Accordingly, the prevalence of the same impairments in siblings of probands is elevated.^10^

Structural (T1‐weighted, T2‐weighted, fluid‐attenuated inversion recovery [FLAIR]) magnetic resonance (MR) imaging in BECTS usually fails to reveal pathological cerebral abnormalities on visual analyses.^11^ Several groups have recently investigated functional connectivity in children with RE.^12^, ^13^, ^14^, ^15^, ^16^, ^17^, ^18^, ^19^, ^20^, ^21^, ^22^ These studies have yielded divergent findings, in terms of directionality (increases or decreases) and localization of alterations in connectivity (Table 1). Familial aggregation, genetics, and neurocognition studies suggest that occult abnormalities might also be identifiable in the siblings of children with BECTS. However, to our knowledge, functional connectivity has not yet been performed in siblings.

**Table 1:** Resting functional MRI connectivity studies in BECTS

Authors (year)	BECTS population (mean years ± SD)	Control population (mean years ± SD)	Selected neuropsychology	Analyses	Findings
Ji et al. (2016)^23^	20 BECTS with IEDs (9 ± 2) 23 BECTS without IEDs (10 ± 2)	28 HC (10 ± 2)	WISC Full‐Scale IQ BECTS with IEDs 112 ± 12 BECTS without IEDs 107 ± 15 HC 114 ± 15	Graph theory with regional parcellation; NBS	*Decreased* global and local efficiency for both BECTS with and without IEDs; *decreased* functional connectivity for both BECTS with and without IEDs in L and R FL and R PL
Luo et al. (2016)^19^	21 BECTS (9 ± 2) (4 subs. excluded)	20 HC (9 ± 2) (3 subs. excluded)	WISC Full‐Scale IQ BECTS 81 ± 12 HC 102 ± 6	Seed‐based correlations; seed‐based GCA	*Increased* functional connectivity within and anticorrelation between DMN and TPN; significant difference in outflow:inflow in the left sup. F and L O
Luo et al. (2015)^22^	21 BECTS (9 ± 2)	20 HC (9 ± 2)	WISC Full‐Scale IQ BECTS 78 ± 12 HC 109 ± 6	Seed‐based correlations	*Decreased* functional connectivity for four regions with increased GMV: R Puta, R Insula/Operc, R SMA and L paracentral lobule
Wu et al. (2015)^15^	32 BECTS (10 ± 2)	25 HC (10 ± 2)	WISC Full‐Scale IQ BECTS 90 ± 8 Controls 100 ± 16	Voxel‐mirrored homotopic connectivity	*Decreased* interhemispheric connectivity in several FL regions and cerebellum
Wu et al. (2015)^16^	37 BECTS (10 ± 2)	25 HC (10 ± 2)	WISC Full‐Scale IQ BECTS 91 ± 9 Controls 101 ± 16	Seed‐based GCA	*Increased* driving from sensorimotor cortex to R med FL, pCingG *Decreased* drive to L IFG
Xiao et al. (2015)^20^	73 BECTS (10 ± 2)	73 HC (10 ± 2)	WISC Full‐Scale IQ BECTS 97 ± 12 HC 102 ± 11	Graph theory with regional parcellation; NBS	*Decreased* global CC and efficiency; *decreased* connectivity in sensorimotor regions
Xiao et al. (2015)^17^	15 BECTS with ADHD (8 ± 2) 15 BECTS without ADHD (9 ± 2)	15 HC (8 ± 2)	WISC Full‐Scale IQ BECTS‐ADHD 102 ± 6 BECTS w/o ADHD 101 ± 5 HC 102 ± 6	Seed‐based correlation	BECTS with ADHD—*decreased* DAN connectivity BECTS w/o ADHD—*increased* DAN connectivity BECTS ± ADHD—*increased* VAN and DMN connectivity
Zeng et al. (2015)^18^	16 new‐onset BECTS (9 ± 2) 17 chronic BECTS (11 ± 2)	18 HC (11 ± 2)	Annual Mandarin School Exam New‐onset BECTS 81 ± 12 Chronic BECTS 76 ± 13 HC 87 ± 9	ReHo	New‐onset BECTS—*decreased* ReHo in cerebellum, DMN, OL; *increased* ReHo in sensorimotor and language regions Chronic BECTS—*decreased* ReHo in cerebellum, DMN, OL; *increased* ReHo in language regions
Besseling et al. (2014)^21^	22 BECTS (11 ± 2)	22 HC (11 ± 2)	Nil	Graph theory with regional parcellation; correlation of structural‐functional graphs	*NAD* for connectivity *Decreased* structural‐functional correlation in medial P and C‐T clusters
Tang et al. (2014)^14^	30 BECTS (10 ± 2)	20 HC (10 ± 2)	WISC Full‐Scale IQ BECTS 110 ± 15 HC 116 ± 17	ReHo	*Increased* ReHo L IFG, L SFG, L and R preCentG, R postCentG gyrus; R AngG, R SMG, L and R sup P *Decreased* L and R orbito‐FL and L and R T pole, R putamen, cerebellum
Besseling et al. (2013)^12^	22 BECTS (11 ± 2)	22 HC (10 ± 2)	CELF‐4 Core Language Score BECTS 95 ± 18 HC 105 ± 11	ICA of task‐evoked data	*Decreased* connectivity between L IFG and “sensorimotor network”
Besseling et al. (2013)^13^	23 BECTS (11 ± 2)	21 HC (10 ± 2)	CELF‐4 Core Language Score BECTS 92 ± 18 HC 106 ± 11	Seed‐based correlation	*Decreased* connectivity between L pre‐ and postCentG and R IFG

ADHD, attention‐deficit/hyperactivity disorder; AngG, angular gyrus; BECTS, benign epilepsy with centrotemporal spikes; C, central; CC, clustering coefficient; CELF, Clinical Evaluation of Language Fundamentals; DAN, dorsal attention network; DMN, default mode network; F, frontal; FL, frontal lobe; G, gyrus; GCA, Granger Causality analysis; GLM, general linear model; GMV, gray matter volume; HC, healthy controls; ICA, independent components analysis; IEDs, interictal epileptiform discharges during functional MRI; IFG, inferior frontal gyrus; L, left/lobe; med, medial; MRI, magnetic resonance imaging; NAD, no abnormality detected; NBS, Network‐Based Statistic; O, occipital; Operc, frontal operculum; PL, parietal lobe; preCentG, precentral gyrus; pCingG, posterior cingulate gyrus; postCentG, postcentral gyrus; Puta, putamen; R, right; ReHo, regional homogeneity; SD, standard deviation; SFG, superior frontal gyrus; SMA, supplementary motor area; SMG, supramarginal gyrus; sub, subsequently; sup, superior; T, temporal; TPN, task‐positive network; VAN, ventral attention network; WISC, Wechsler Intelligence Scale for Children.

Seed‐based connectivity studies have often been limited to examination of the connectivity of a few regions of interest,^13^ whereas independent components analysis has been used to identify a specific network of interest.^12^ There have been few attempts to study whole‐brain, functional network connectivity in BECTS using regional parcellation.^20^, ^21^, ^23^

Graph theory is a mathematical framework that allows the quantification of topological characteristics (i.e., architecture) of complex functional brain networks. Xiao et al.^20^ found that participants with BECTS exhibited both global and local alterations in network architecture. In contrast, graph analysis failed to reveal abnormalities of functional connectivity in another study.^21^

The Network Based Statistic (NBS^24^) is a nonparametric method of family‐wise error rate control analogous to cluster‐based thresholding of parametric maps, which allows comparison of the connectivity measures (edges) themselves. Using NBS, participants with BECTS were shown to have decreased connectivity in two distinct subnetworks; a six‐node “sensorimotor” network, which included the left rolandic operculum and the postcentral gyri, and a four‐node posterior network involving the fusiform gyri and occipital lobes.^20^

In the present study, we compared functional connectivity between children with BECTS, siblings, and healthy controls. The primary objective was to test the hypothesis that patients with BECTS had abnormal network architecture and subnetwork functional connectivity. The secondary objective was to test the hypothesis that siblings share the same pattern of functional connectivity abnormalities as BECTS probands.

## Methods

### Ethical approvals and consent

The study was approved by London Camberwell‐St. Giles National Health Service Research Ethics Committee (10/H0807/93). Because all participants were younger than 16 years of age, informed, written consent was obtained from a parent of each, along with assent from the participants themselves.

### Study design

We conducted a cross‐sectional comparison of patients with BECTS, siblings of patients with BECTS, and healthy control participants.

### Epilepsy, sibling, and control samples

Twenty‐six right‐handed patients with BECTS (Rolandic epilepsy) were recruited from outpatient clinics in southeast England between October 2012 and October 2014. One patient was subsequently excluded because of the significant head motion during the functional MR imaging. Demographics of the final cohort are listed in Table 2. Their diagnoses were based on history, seizure semiology (e.g., unilateral orofacial or upper extremity sensorimotor symptoms; anarthria; secondarily generalized seizures), interictal electroencephalography (EEG), and MR imaging data (where available). Exclusion criteria included interictal interval of 1 year or more, claustrophobia, and standard MR contraindications. The median epilepsy duration was 34 months (range 10–169 months); the median interictal interval was 2 months (range 0–9 months). Centrotemporal epileptiform discharges were predominantly left‐sided in 9 patients, right‐sided in 12 patients, and bilateral in 4 patients. Thirteen patients were taking antiepileptic drugs (AEDs).

**Table 2:** Demographics and motion indices after exclusions

	BECTS (n = 25) Mean (SD)	Siblings (n = 12) Mean (SD)	Healthy controls (n = 20) Mean (SD)
Age	10.6 (1.6)	11.6 (2.5)	11.9 (1.5)
Males:females	16:9	4:8	12:8
Relative RMS (mm)	0.008 (0.008)	0.006 (0.007)	0.007 (0.007)

BECTS, benign epilepsy with centrotemporal spikes; RMS, root mean square, a measure of movement during the acquisition of the resting functional MRI data; SD, standard deviation.

Fourteen right‐handed siblings of patients with BECTS were recruited, but two subsequently withdrew prior to scanning. The BECTS proband did not meet the BECTS group age inclusion criterion (7–14 years) for three members of the Sibling group (Table 2); these participants did not, therefore, have a family member in the BECTS group. One of the siblings is known to have had two (presumed) seizures only; she was subsequently diagnosed with BECTS (Rolandic epilepsy) after sleep electroencephalography. Her most recent seizure occurred approximately 2 years prior to the MR scan. Exclusion of this participant does not alter the conclusions of this study. EEG data was available for 6 of the 11 remaining siblings; epileptiform discharges were seen for 1 sibling only (maximal T4 electrode).

A control group of 24 right‐handed healthy volunteers was recruited through advertisement. The healthy controls (HCs) were without history of neurological or psychiatric disorder (according to parental reports). Four healthy controls were subsequently excluded to improve age matching; following these exclusions there were no significant differences in age or gender between groups.

### FMRI data acquisition

One hundred eighty‐four gradient echo planar imaging (EPI) volumes were acquired on a 3‐Tesla Signa HDx scanner (GE Healthcare, Little Chalfont, Buckinghamshire, United Kingdom) at the Centre for Neuroimaging Sciences (Institute of Psychiatry, Psychology & Neuroscience, King's College London, United Kingdom) with the following parameters: TE = 25 ms, TR = 2,000 ms; flip angle = 75°; slice thickness = 2.4 mm, slice gap = 1 mm, spatial (axial) positions = 38 (prescribed parallel to the AC‐PC line), matrix = 64 × 64, field of view 24.0 cm.

### Psychometrics

All participants completed the Wide Range Achievement Test (WRAT‐4^25^). The majority of participants also completed the following: the Wechsler Abbreviated Scale of Intelligence (WASI^26^); the Children's Communication Checklist (CCC, Second Edition^27^); the SCAN (Adolescent and Adult/Child versions; auditory processing^28^, ^29^) the Test of Word Reading Efficiency (TOWRE‐2^30^); the Conner's (Parent) Rating Scale (Revised^31^); the Strengths and Difficulties Questionnaire (SDQ; http://www.sdqinfo.com/); and the Developmental Coordination Disorder Questionnaire (DCD‐Q‐07; http://dcdq.ca/). Full‐scale Intelligence Quotient (IQ) data was based on two subtests (Vocabulary and Matrix Reasoning), rather than four, for one participant with BECTS.

### MRI analyses

#### fMRI preprocessing

We preprocessed the functional magnetic resonance imaging (fMRI) data using tools from both the FSL (FSL, version 4.1.7; http://fsl.fmrib.ox.ac.uk) and SPM (version 12; Functional Imaging Laboratory, http://www.fil.ion.ucl.ac.uk/spm/software/spm12/) software.

Because significant intervolume head motion was evident in many datasets, particularly toward the end of the scan, volumes 4–156 were used for the analyses. Motion outliers were identified for each participant separately using the fsl_motion_outliers script, using the default motion metric, which is known as “refmse.” The mean squared error (MSE) of intensity differences between each volume and the reference (middle) volume was computed. Each volume with an MSE of greater than the (within‐subject) 75th percentile plus 1.5 times the interquartile range of these measures was classified as an outlier.

Across participants, the 90th percentile of the number of outliers in each unprocessed 152‐volume dataset was 17.4 (which we have rounded to 18). Accordingly, outlier volumes were replaced sequentially by the remaining volumes (157 onward) until the total number of outliers was <18.

Correction for intervolume motion was performed using SPM by realignment of all fMRI volumes to the first volume. The relative root mean square (rRMS) movement over each run was calculated from the motion regressors, and participants with a relative root mean square >0.035 mm (similar to cut‐offs used in other studies involving children) were excluded from further analyses.

The time‐series data were bandpass filtered with a high‐pass and low‐pass of 0.01 and 0.10 Hz, respectively. Correction for intravolume acquisition delay was performed using SPM12. The images were then spatially normalized to the SPM EPI template in Montreal Neurological Institute standard space (voxel size 2 × 2 × 2 mm) by linear followed by nonlinear registration (16 iterations, 7 × 9 × 7 basis functions).

The mean cerebrospinal fluid (CSF) and white matter signals were extracted from each dataset using the SPM canonical templates after thresholding at >0.80 and >0.90, respectively. The time‐series fMRI data were smoothed with an isotropic Gaussian filter (5 mm full‐width at half maximum). Residual images were generated using a General Linear Model (GLM) in FSL, with the mean CSF and mean white matter signals, the six realignment parameters, and the motion outliers regressed as nuisance variables.

#### Graph construction

The mean times series of each of 90 anatomical regions of interest (ROIs) were extracted from each residual image using the Automated Anatomical Labeling atlas.^32^ Functional connectivity was quantified as pairwise Spearman's rank correlation coefficients, resulting in 90 × 90 adjacency matrices. Negative correlation coefficients were multiplied by −1, that is, the absolute value of the coefficient was used (Fig. S1). Sparse, undirected graphs were constructed by proportional thresholding of the (weighted) matrices at 0.20–0.38 (i.e., connection density 20–38%, intervals of 0.02), followed by binarization (Fig. S1), using the Brain Connectivity Toolbox (https://sites.google.com/site/bctnet/; Rubinov and Sporns^33^), running in MATLAB (version 7.8 2009a; MathWorks). Proportional thresholding yields graphs of identical connection density across participants, which is preferable for comparisons of topology.

#### Graph metrics

The global efficiency (E), local efficiency (E_loc_), mean local efficiency (MeanE_loc_), and nodal degree (d) were calculated for each graph and subsequently compared between groups. For further details, please see Appendix S1.

### Statistical analyses

#### Psychometrics

We used the univariate ANOVA to compare the following between groups: WASI Full‐Scale IQ, Verbal IQ, Performance IQ; CCC‐2 General Communication Composite (GCC); TOWRE‐2 Total Word Reading Efficiency (TWRE), Sight Word Efficiency (SWE), and Phonemic Decoding Efficiency (PDE); WRAT‐4 Reading Composite, Word Reading, Sentence Comprehension, and Spelling; and SDQ Hyperactivity.

We used the Kruskal–Wallis test to compare the following metrics, which significantly deviated from the normal distribution, between groups: the absolute difference between WASI verbal and performance IQ; Conner's Global Impairment (GI) total T score and ADHD Index T score; DCD‐Q‐07 Total; Scan A/C Competing Words standard score; SDQ Total Disability, Emotional Symptoms, Conduct Problems, and Peer Problems.

#### Motion

The relative mean root mean square (i.e., motion) was compared between groups using the Kruskal–Wallis test, because the data significantly deviated from the normal distribution.

#### Subnetwork connectivity

We used permutation testing (50,000 permutations) as implemented in the Network‐Based Statistics (NBS) toolbox (version 1.2) to identify subnetworks that differed significantly in connectivity (i.e., correlation coefficients) between groups using t tests (BECTS vs. controls, siblings vs. controls, BECTS vs. siblings), with age as a covariate, gender as an explanatory variable, and NBS^24^ correction for multiple comparisons. The test statistic threshold is an arbitrary threshold applied after mass univariate testing of each connection, but prior to the cluster‐based analysis in topological space. We employed several thresholds (3.0, 3.2, 3.4, and 3.6) in order to identify alterations in subnetwork connectivity that were not contingent on thresholding. Component (i.e., subnetwork) size was defined by intensity (i.e., the sum of test statistic values across all connections of the component).

We quantified the association between median absolute connectivity within the (BECTS < controls) subnetwork identified at the most conservative test statistic (3.6) and selected psychometrics separately for BECTS and control groups, using Spearman's rank correlation coefficient. Based on the spatial distribution of the resultant subnetwork and availability of the data, we selected the WRAT‐4 subtests (Table 3). We also quantified the relationship of the median absolute connectivity and the duration of epilepsy. We employed false discovery rate (FDR) correction (q = 0.05) for seven comparisons.

**Table 3:** Psychometrics for the groups

	BECTS mean (SD), Cohen's d	Siblings mean (SD), Cohen's d	Healthy controls mean (SD)	(df, error); F, p value
Intelligence				
**WASI Full Scale IQ** (n = 24; 12; 20)	109.5 (13.4), 0.4	105.0 (8.0), 1	114.8 (12.5)	(2, 53); 2.6, 0.09
WASI Verbal IQ (n = 24; 12; 20)	109.8 (12.5), 0.1	102.0 (10.7), 0.7	111.3 (14.5)	(2, 53); 2.1, 0.14
WASI Performance IQ (n = 24; 12; 20)	107.0 (16.0), 0.6	107.2 (11.2), 0.7	115.1 (10.5)	(2, 53); 2.4, 0.10
WASI Verbal IQ – Performance IQ absolute difference (n = 23; 12; 20)	12.4 (10.0), 0.2	13.1 (10.9), 0.3	10.1 (8.6)	Kruskal–Wallis p = 0.59
Communication and language				
**CCC‐2 General Communication Composite** (n = 23; 11; 19)	72.3 (24.8), 0.9	80.0 (20.4), 0.7	90.5 (10.8)	(2, 50); **4.3, 0.02** *
**SCAN A/C Competing Words** (n = 24; 11; 18)	90.5 (19.1), 0.3	90.3 (19.1), 0.2	85.7 (19.1)	Kruskal–Wallis p = 0.74
**TOWRE‐2 Total Word Reading Efficiency** (n = 24; 12; 18)	100.1 (13.4), 0.4	97.6 (11.9), 0.7	106.2 (12.4)	(2, 51); 1.9, 0.15
TOWRE‐2 Sight Word Efficiency (n = 24; 12; 18)	97.3 (13.8), 0.4	97.3 (12.5), 0.4	103.2 (13.8)	(2, 51); 1.1, 0.33
TOWRE‐2 Phonemic Decoding Efficiency (n = 24; 12; 18)	102.7 (13.5), 0.4	98.3 (11.6), 0.9	107.9 (10.6)	(2, 51); 2.4, 0.10
**WRAT‐4 Reading Composite** (n = 25; 12; 20)	108.3 (14.2), 0.4	111.3 (13.7), 0.2	113.8 (10.2)	(2, 54); 1.0, 0.37
WRAT‐4 Word Reading (n = 25; 12; 20)	107.8 (13.9), 0.4	111.2 (17.4), 0.1	112.6 (9.8)	(2, 54); 0.7, 0.48
WRAT‐4 Sentence Comprehension (n = 25; 12; 20)	108.4 (15.2), 0.4	111.1 (9.7), 0.3	114.2 (13.4)	(2, 54); 1.0, 0.37
**WRAT‐4 Spelling** (n = 24; 12; 20)	112.5 (19.2), 0.2	113.3 (17.6), 0.1	115.4 (14.5)	(2, 53); 0.2, 0.86
Behavioral symptoms				
**Conner's Global Impairment** (n = 23; 11; 17)	109.2 (20.1), 0.7	99.5 (15.6), 0.2	97.2 (14.9)	Kruskal–Wallis **p = 0.02** *
**Conner's Attention Deficit Hyperactivity Disorder Index** (n = 23; 11; 17)	107.9 (20.5), 0.4	100.7 (13.4), 0.1	99.6 (17.6)	Kruskal–Wallis p = 0.23
**SDQ Total Disability** a (n = 25; 12; 18)	10.1 (4.8), 0.9	5.8 (5.7), 0.0	5.6 (5.2)	Kruskal–Wallis **p = 0.004** **
SDQ Emotional Symptoms (n = 25; 12; 18)	2.4 (1.9), 1.1	0.8 (0.9), 0.1	0.7 (0.7)	Kruskal–Wallis **p = 0.002** **
SDQ Conduct Problems (n = 25; 12; 18)	1.5 (1.5), 0.2	1.0 (1.1), 0.2	1.3 (2.0)	Kruskal–Wallis p = 0.51
SDQ Hyperactivity (n = 25; 12; 18)	4.5 (2.9), 0.8	2.8 (2.7), 0.1	2.5 (2.2)	(2, 52); 3.5, 0.04
SDQ Peer Problems (n = 25; 12; 18)	1.7 (1.8), 0.3	1.3 (2.1), 0.1	1.2 (1.4)	Kruskal–Wallis p = 0.36
Coordination				
**DCD‐Q‐07 Total** b (n = 24; 10; 19)	56.9 (14.5), 1.0	66.2 (9.3), 0.4	68.9 (7.2)	Kruskal–Wallis **p = 0.005** **

Lower scores indicate greater deficit, other than for behavioral symptoms scales.

Bold font denotes results that remain significant after correction for multiple comparisons by FDR (*q = 0.10; **q = 0.05).

a The SDQ Total Disability score has a maximum of 40 (each subtest has a maximum score of 10).

b The DCD‐Q‐07 Total has a maximum score of 75. Scan A/C scores, Conner's Global Impairment total t scores, and Conner's Attention Deficit Hyperactivity Disorder total t scores were converted to standard scores (distribution mean = 100 and standard deviation = 15).

BECTS, benign epilepsy with centrotemporal spikes; CCC, Children's Communication Checklist; DCD‐Q‐07, Developmental Coordination Disorder Questionnaire; df, degrees of freedom; FDR, false discovery rate; IQ, Intelligence Quotient; SD, standard deviation; SDQ, Strengths and Difficulties Questionnaire; TOWRE, Test of Word Reading Efficiency; WASI, Wechsler Abbreviated Scale of Intelligence; WRAT, Wide Range Achievement Test.

#### Pairwise connectivity

We used permutation testing (50,000 permutations) as implemented in the NBS toolbox to identify pairwise difference in connectivity between groups using t tests (BECTS vs. controls, siblings vs. controls, BECTS vs. siblings), with age as a covariate, gender as an explanatory variable, and FDR (q = 0.05) correction for 4,005 tests (90 nodes × 89 nodes × 0.5).

## Results

### Psychometrics

There were significant differences in CCC‐2 General Communication Composite (BECTS < siblings < controls), Conner's Global Impairment (BECTS > siblings > controls), SDQ Total Disability (BECTS > siblings > controls) and Emotional Symptoms (BECTS > siblings > controls), and DCD‐Q‐07 Total (BECTS < siblings < controls) between groups (FDR correction, q = 0.10; Table 3). Post hoc tests revealed a significant difference between BECTS and control groups in CCC‐2 General Communication Composite (p = 0.02 after Bonferroni correction), Conner's Global Impairment (p = 0.02 after Bonferroni correction); significant differences between BECTS and control groups and also between BECTS and sibling groups in SDQ Total Disability (p = 0.006 and p = 0.04 after Bonferroni correction, respectively); and SDQ Emotional Symptoms (p = 0.003 and p = 0.03 after Bonferroni correction, respectively); and between BECTS and control groups in DCD‐Q‐07 Total (p = 0.006 after Bonferroni correction).

### Comparison of motion

The rRMS for each group is provided in Table 2. There were no significant differences in rRMS between groups (Kruskal–Wallis test, p = 0.92).

### Graph metrics

There were no significant differences in any of the graph metrics between groups (Figs. S2 and S3).

### Subnetwork connectivity

**Figure 1:**
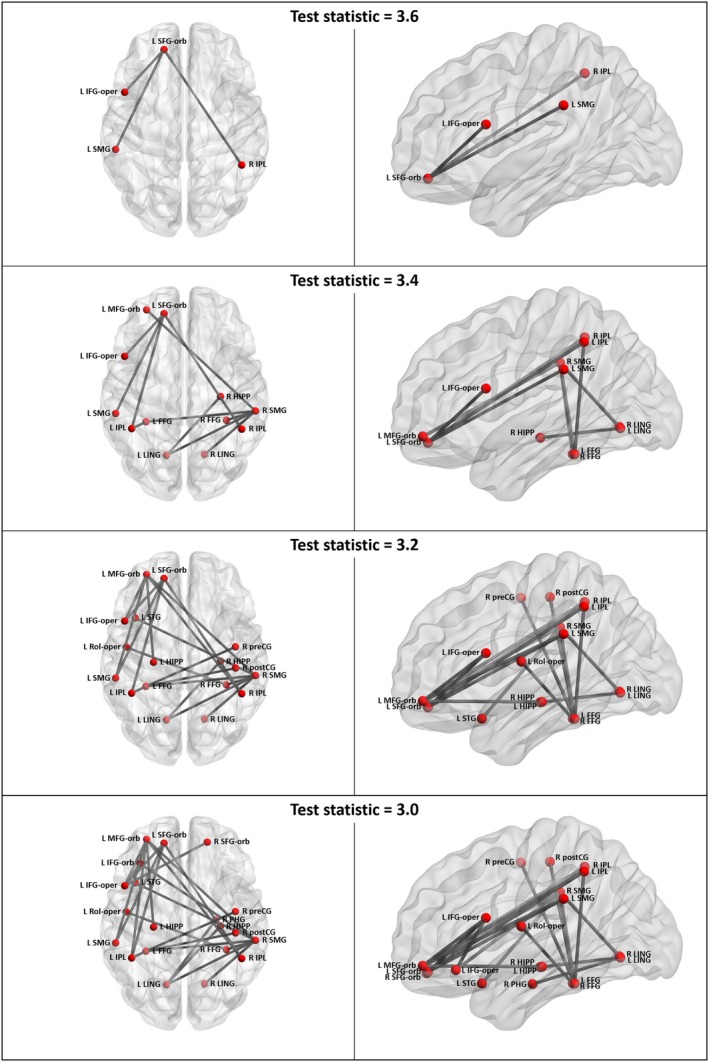
Subnetworks of decreased functional connectivity in participants with BECTS relative to controls. FFG, fusiform gyrus; HIPP, hippocampus; IFG‐orb, inferior frontal gyrus, orbital part; IFG‐oper, inferior frontal gyrus, opercular part; IPL, inferior parietal lobe excluding supramarginal and angular gyri; L, left; LING, lingual gyrus; MFG‐orb, middle frontal gyrus, oirbital part; PHG, parahippocampal gyrus; postCG, postcentral gyrus; preCG, precentral gyrus; R, right; Rol‐oper, rolandic operculum; SFG‐orb, superior frontal gyrus, orbital part; SMG, supramarginal gyrus; STG, superior temporal gyrus. Figure was prepared using BrainNet Viewer.^42^

**Figure 2:**
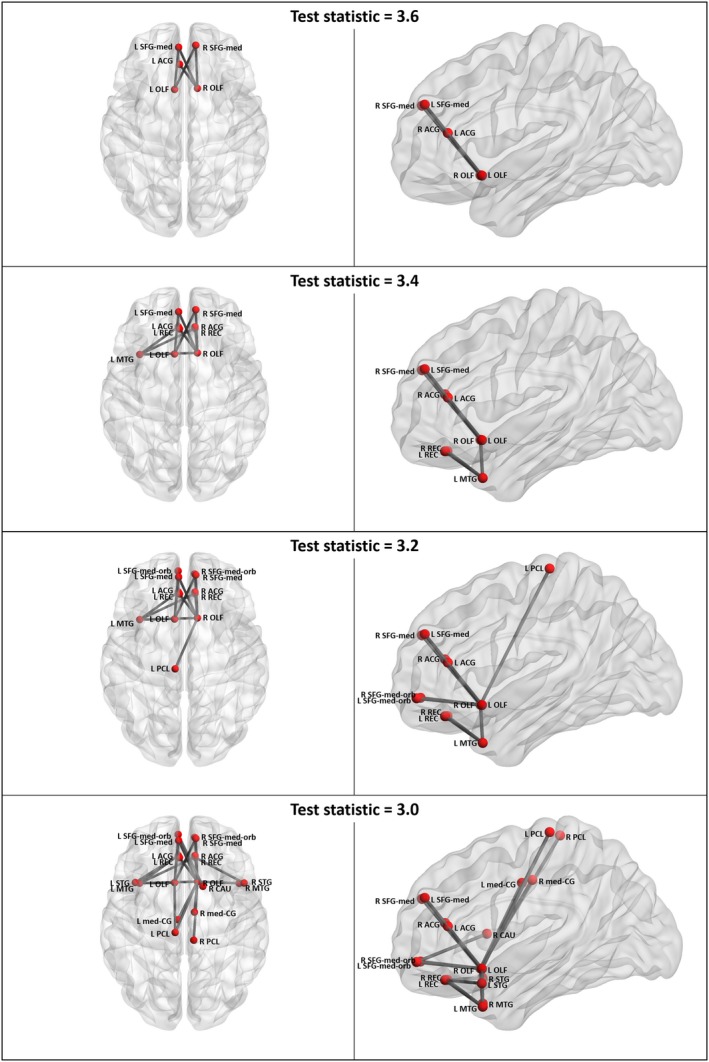
Subnetworks of increased functional connectivity in participants with BECTS relative to controls. ACG, anterior cingulate gyrus; CAU, caudate nucleus; L, left; med‐CG, median cingulate and paracingulate gyrus; MTG, middle temporal gyrus; OLF, olfactory cortex; PCL, paracentral lobule; R, right; REC, gyrus rectus; SFG‐med, superior frontal gyrus, medial part; SFG‐med‐orb, superior frontal gyrus, medial orbital part; STG, superior temporal gyrus. Figure was prepared using BrainNet Viewer.^42^

Relative to controls, participants with BECTS had a significant decrease in connectivity (p < 0.05) within a four‐node, three‐edge subnetwork that included the left superior frontal gyrus—orbital part, the left inferior frontal gyrus—opercular part, the left supramarginal gyri, and the right inferior parietal lobe (test statistic = 3.6, p = 0.04; Fig. 1). Decreased connectivity was also observed in similar but more extensive subnetworks at test statistic thresholds of 3.4 (12 nodes, 11 edges; p = 0.03), 3.2 (17 nodes, 19 edges; p = 0.04), and 3.0 (20 nodes, 24 edges; p = 0.047). A significant increase in connectivity was identified in a five‐node, five‐edge subnetwork that included the left and right superior medial frontal regions, the left and right olfactory regions, and the left anterior cingulate gyrus (test statistic = 3.6, p = 0.04; Fig. 2), and in similar but more extensive subnetworks at test statistics of 3.4, 3.2, and 3.0.

Controlling for age and gender by General Linear Model, connectivity within the four‐node (BECTS < controls) subnetwork was as follows: BECTS group estimated marginal mean 0.27, standard deviation 0.24, Cohen's d versus healthy controls 1.3; siblings group 0.42 ± 0.24, 0.7; healthy controls group 0.58 ± 0.23. There were no significant correlations between median connectivity within the subnetwork and WRAT‐4 subtests for the BECTS group (p ≥ 0.98 FDR correction) or with epilepsy duration (p = 0.20), whereas for controls there was a positive correlation between median connectivity within the subnetwork and WRAT‐4 Sentence Comprehension (ρ = 0.58, p = 0.049 FDR correction).

There were no significant differences in subnetwork connectivity between siblings and healthy controls or between BECTS and siblings groups.

### Pairwise connectivity

Relative to controls, the BECTS group had a significant decrease in connectivity (p < 0.05) between the left superior frontal gyrus—orbital part and the left inferior frontal gyrus—opercular part, and also the left supramarginal gyri, and a significant increase in connectivity between the right olfactory region and the left and right superior medial frontal regions.

Relative to siblings, the BECTS group had no significant decreases or increases in connectivity.

There were no significant differences in connectivity between the siblings and control groups.

## Discussion

The present study shows that participants with BECTS have a significant decrease in functional connectivity relative to controls within a subnetwork that included, at a minimum, the left inferior frontal gyrus (opercular part), the left supramarginal gyrus, and the right inferior parietal lobe.

Tomasi and Volkow^34^ identified a reproducible resting fMRI “language network” derived from 970 healthy controls, which included the left inferior frontal gyrus—opercular part (Broca's area), the left supramarginal gyrus (Wernicke's area), and the inferior aspect of the right parietal lobe. In the present study, participants with BECTS had a decrease in connectivity within a four‐node, three‐edge subnetwork that substantially overlaps with this language network. At lower test statistics, we identified decreases in additional components of the language network, such as the left middle frontal gyrus, the left inferior frontal gyrus, orbital part, the left superior temporal gyri, and the parietal lobes lobe.^34^

The left inferior frontal gyrus—opercular part comprises a subregion of the left inferior frontal gyrus, lesions of which are associated with Broca's (expressive) aphasia. This subregion has been implicated in auditory processing, speech comprehension, covert articulatory planning, and auditory and motor feedback during speech production.^35^

Transcranial magnetic stimulation studies implicate the supramarginal gyri in phonological processing and visual word recognition.^36^ The decreases in functional connectivity seen in the left rolandic operculum, and in the right pre‐ and postcentral gyri may be associated with the centrotemporal spikes/shape waves (CTS) that typify BECTS and that have been consistently localized to this region.^37^ It is unsurprising that both left and right sensorimotor regions were implicated, given the epileptiform discharges observed in our BECTS group. In addition to its role in overt articulation, the precentral gyrus has, via neuroimaging, been implicated in sublexical reading (reviewed in Ref. 35).

Together, therefore, our BECTS probands had a significant decrease in functional connectivity between several key mediators of speech processing, language, and reading, constituents of the “language network.”^34^ The children with BECTS scored markedly lower (Cohen's d = 0.9) than controls on the CCC‐2 General Communication Composite. Reading scores were also moderately decreased for the patient group relative to controls (e.g., TOWRE‐2 TWRE d = 0.4; WRAT‐4 Reading Composite d = 0.4), although these differences did not reach statistical significance. These data suggest multidomain language and literacy impairment, consistent with the findings of our recent meta‐analysis.^3^

In the present study, connectivity within the four‐node subnetwork was not significantly correlated with WRAT‐4 literacy psychometrics in the BECTS group (but was in healthy controls). Other investigators have, on occasion, failed to demonstrate a correlation between functional connectivity and attention or language metrics in RE.^17^, ^20^ Our BECTS population appears to have suffered less severe cognitive and language impairment than those of previous studies.^13^, ^19^, ^20^ The lack of correlation could reflect measurement error, the recruitment bias toward high‐performing patients (who may have adaptive, compensatory mechanisms), or the influence of an unstudied confounder. It remains a challenge to demonstrate that decreased functional connectivity *contributes* to the pathophysiology of coexistent cognitive impairment in BECTS; future studies in children with BECTS should quantify the association between enhancement of connectivity, for example, via an fMRI‐mediated neurofeedback task (for review, Ref. 38) or an off‐line training program,^39^ and improvement in literacy and language skills.

A task‐driven fMRI study suggested that language lateralization is delayed in BECTS.^40^ It was also recently demonstrated, albeit in adults, that functional connectivity can predict language laterality.^41^ Therefore, the left hemispheric decreases in functional connectivity we identified in our children with BECTS might reflect delayed left‐lateralization of language processing; this hypothesis could be tested by longitudinal follow‐up.

Our finding of decreased functional connectivity in children with BECTS is broadly consistent with earlier studies.^12^, ^13^, ^14^, ^15^, ^18^, ^20^, ^22^, ^23^ In the largest (n = 73 per group) and most comparable study, the NBS method revealed that children with BECTS had decreased connectivity in two subnetworks, including the left (Rolandic) operculum, interictal epileptic discharge (IED)‐generating regions (bilateral postcentral gyrus), fusiform gyri, and occipital lobes;^20^ these data are consistent with our findings. Using graph theory, the authors also identified a decrease in the centrality of the right supramarginal gyrus, at every test statistic other than the most conservative one. The right supramarginal gyrus was also one of the regions identified in our subnetworks.

The identification of the operculum in our subnetwork adds to mounting evidence for decreased connectivity of the left inferior frontal gyrus in BECTS. Decreased resting functional connectivity between this region and the sensorimotor network (including left precentral gyrus) was demonstrated using independent components analysis^12^ and Granger causality analysis.^16^

Although increases in resting functional connectivity have been associated with BECTS (Table 1), two of the studies investigated regional homogeneity (ReHo) rather than low‐frequency fluctuation in BOLD signal,^14^, ^18^ and a third compared the amplitude of low‐frequency fluctuations (ALFF^16^). These studies are not directly comparable to the present work. However, *increases* in medium‐long‐range resting functional connectivity have also been reported in RE.^17^ Where patients with BECTS had co‐occurring attention‐deficit/hyperactivity disorder (ADHD) symptoms, decreases in connectivity were seen,^17^ reflecting our predominant findings in a similar cohort (e.g., 10 participants with BECTS had a “borderline” or “abnormal” score on SDQ Hyperactivity/Inattention). Recently, Luo et al.^19^ identified several increases in functional connectivity that included the left and right superior frontal gyri, as seen in the present study. It is plausible that aberrant maturation leads to both decreases and increases in functional connectivity in different subnetworks and over differing ranges. Longitudinal studies are required to delineate the sequence of functional connectivity alterations in BECTS and their relationship to language lateralization.

### Methodological considerations

Approximately half of the children with BECTS were medicated with an AED, namely, carbamazepine, levetiractetam, lamotrigine, oxcarbazepine, or valproic acid. A pharmacological effect on functional connectivity cannot be excluded; this limitation is common to many epilepsy studies but has seldom been investigated, particularly in children. In the present study, subgroup analyses based on AEDs were not performed because they would have lacked of statistical power.

Intrascan head motion is known to confound measurement of functional connectivity and remains a significant challenge when studying children with epilepsy,^22^ attention deficits, or hyperactivity. We took great care to minimize the influence of motion via (1) exclusion of outlier volumes; (2) image realignment; (3) exclusion of participants with rRMS > 0.035 mm; and (4) regression of realignment parameters. There was no significant difference in rRMS between groups.

We were unable to replicate the recent findings of differences in functional connectivity graph metrics between children with BECTS and controls.^20^, ^23^ This was also the case for a graph study that was similar in size to the current work.^21^ Our results suggest that graph topology metrics are less sensitive to the subtle abnormalities of functional connectivity in BECTS than NBS subnetwork connectivity analyses, perhaps because the abnormal edges constitute too small a proportion of the entire graph.

Our study may not have been sufficiently powered to detect differences between siblings and healthy controls, who (based on several psychometrics) might be hypothesized to show less marked decreases in connectivity. We did observe a decrease in median four‐node subnetwork connectivity for siblings, of a lesser extent than that seen in the BECTS group. Although this did not reach statistical significance, the effect size was large (Cohen's d = 0.7), suggesting low study power. Further investigation of functional connectivity in siblings is warranted.

## Conclusion

Our findings add to mounting evidence for decreased functional connectivity between several key mediators of speech processing, language, and reading in children with BECTS and communication difficulties. Similar decreases appear likely to be present in their siblings. We hypothesize that these decreases reflect delayed lateralization of the language network and contribute to impairment. Longitudinal studies are required to delineate the sequence of functional connectivity alterations in BECTS and examine the effect of enhancement of connectivity on literacy and language skills.

## Disclosure of Conflict of Interest

CJM has received support from GE Healthcare for an unrelated project. GJB receives honoraria from GE Healthcare for teaching and acts as a consultant for IXICO. The remaining authors have no conflicts of interest. We confirm that we have read the Journal's position on issues involved in ethical publication and affirm that this report is consistent with those guidelines.

## Supporting information


**Figure S1.** Mean adjacency matrices (left panel) and graphs (middle and right panels) for participants with BECTS (top), siblings (middle). and healthy controls (bottom).
**Figure S2.** Global efficiency (E) versus connection density, for the BECTS, siblings, and control groups.
**Figure S3.** Mean local efficiency (E_loc_) versus connection density, for the BECTS, siblings, and control groups.

**Appendix S1.** Comparison of graph metrics between groups.
