# Supplementary material for: Decreased functional connectivity within a language subnetwork in benign epilepsy with centrotemporal spikes
Source: Epilepsia Open. 2017 Apr 27;2(2):214–25. doi: 10.1002/epi4.12051 (PMC5719846; doi:10.1002/epi4.12051)
Supplement: Supplementary file 1 — Figure S1. Mean adjacency matrices (left panel) and graphs (middle and right panels) for participants with BECTS (top), siblings (middle). and healthy controls (bottom). Figure S2. Global efficiency (E) versus connection density, for the BECTS, siblings, and control groups. Figure S3. Mean local efficiency (Eloc) versus connection density, for the BECTS, siblings, and control groups. [file EPI4-2-214-s001.docx]

Decreased functional connectivity within a language subnetwork in benign epilepsy with centrotemporal spikes: Supporting Information

**Graph construction**

**
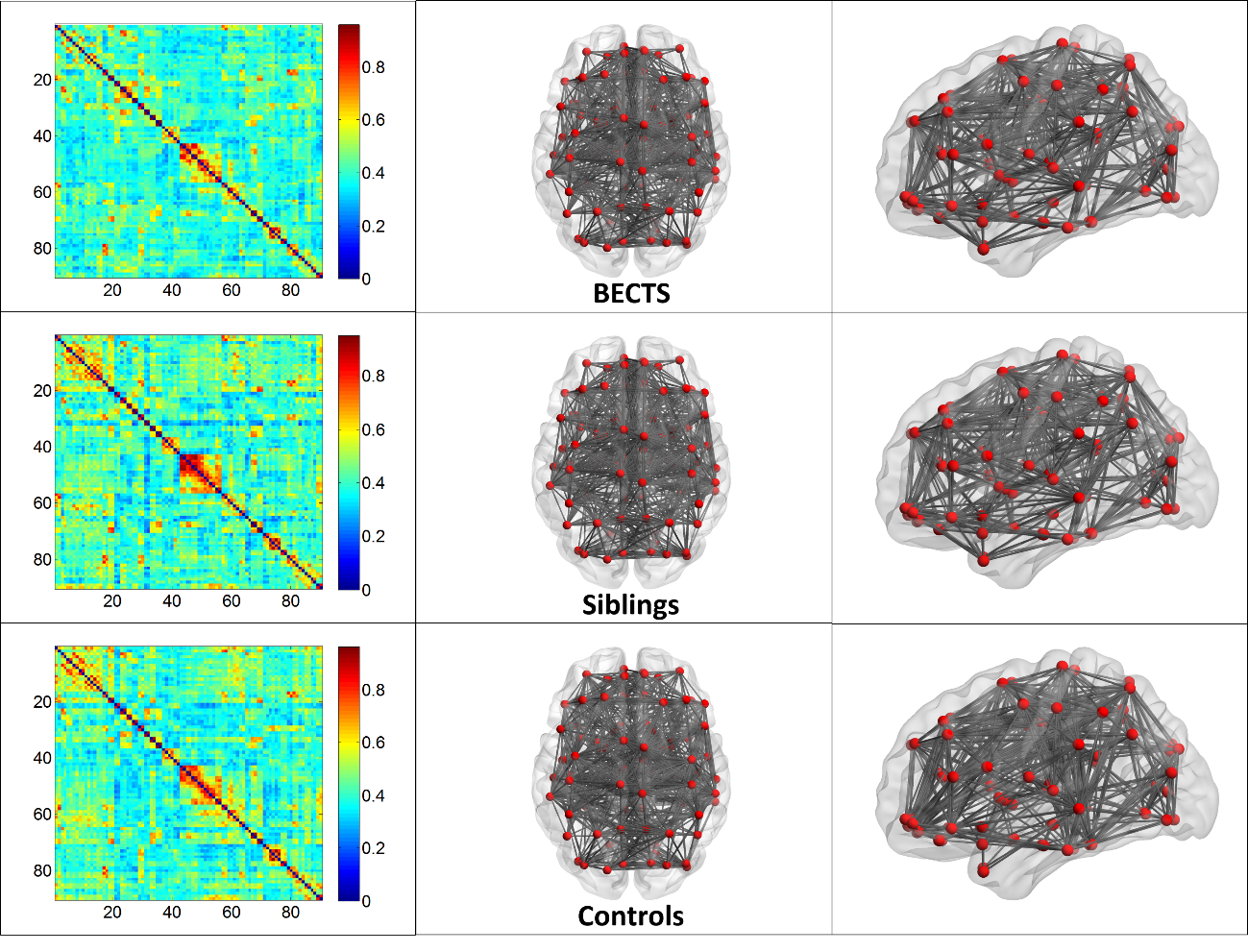
Figure 1: Mean adjacency matrices (left panel) and graphs (middle and right panels) for participants with BECTS (top), siblings (middle) and healthy controls (bottom).** The colourbar indicates the value of Spearman’s correlation coefficient. Graphs are displayed at 20% connection density. Figure was prepared using BrainNet Viewer[^1^](#_ENREF_1).

**
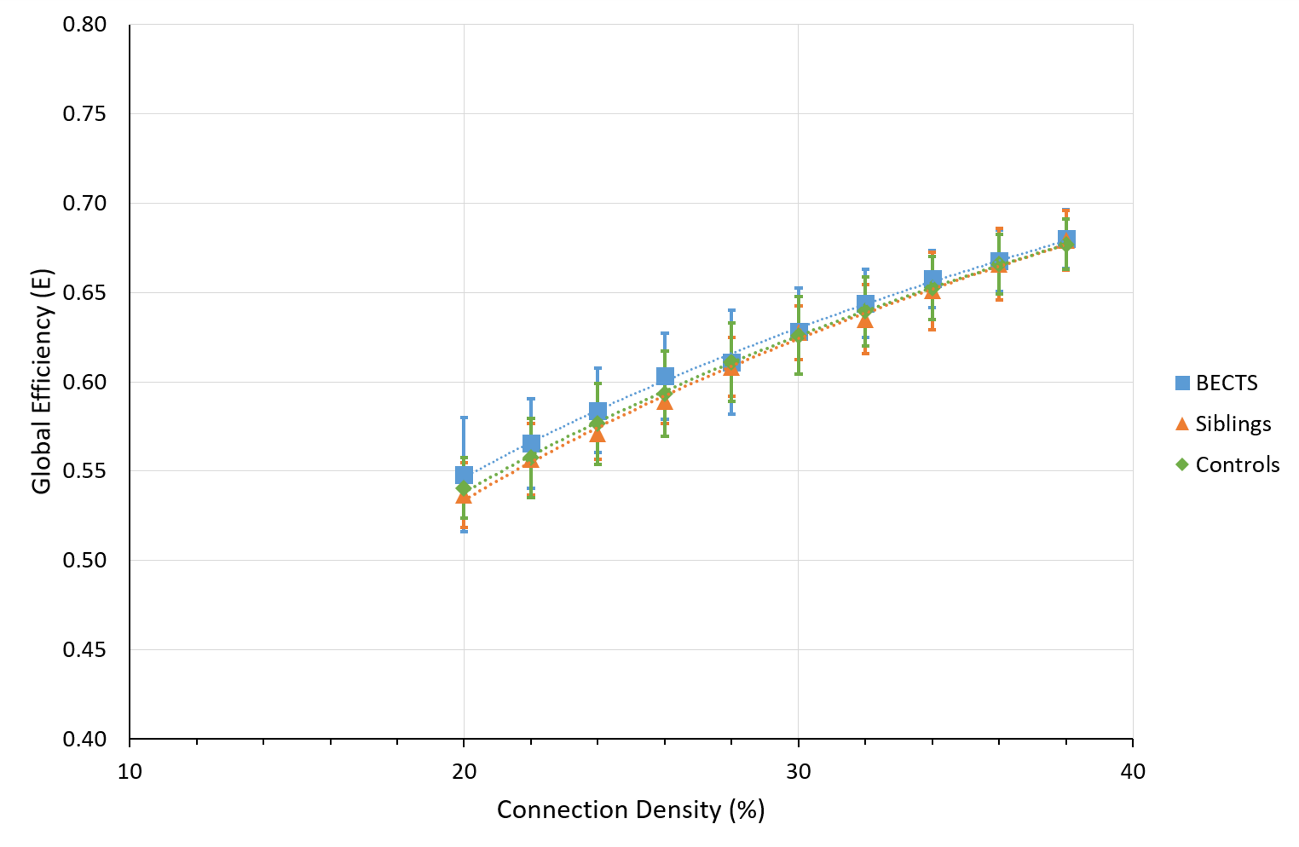
**

**Figure 2: Global efficiency (E) versus connection density, for the BECTS, siblings, and controls groups.**

**
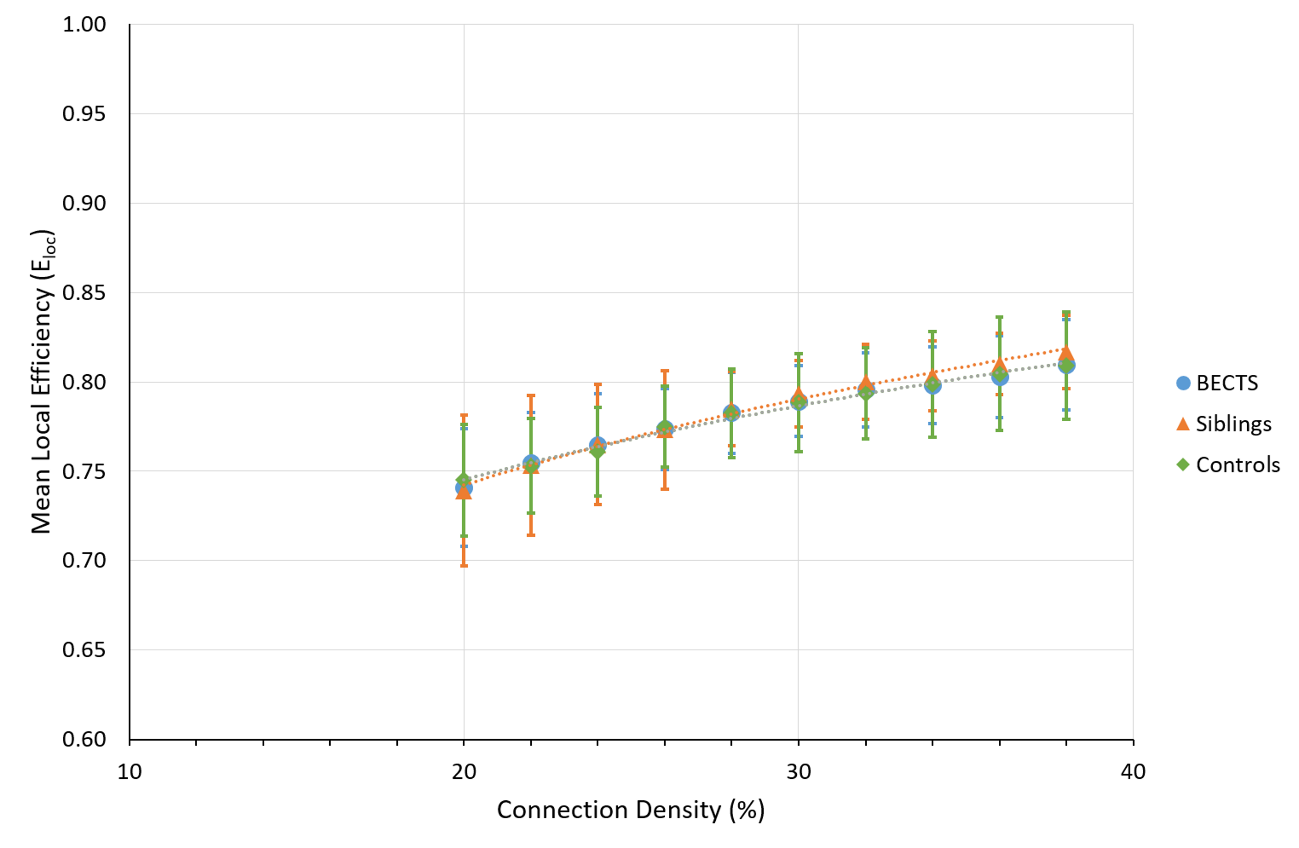
**

**Figure 3: Mean local efficiency (E_loc_) versus connection density, for the BECTS, siblings, and controls groups.**
