# Supplementary material for: Decreased functional connectivity within a language subnetwork in benign epilepsy with centrotemporal spikes
Source: Epilepsia Open. 2017 Apr 27;2(2):214–25. doi: 10.1002/epi4.12051 (PMC5719846; doi:10.1002/epi4.12051)
Supplement: Supplementary file 2 — Appendix S1. Comparison of graph metrics between groups. [file EPI4-2-214-s002.docx]

Appendix S1: Comparison of graph metrics between groups

**Global metrics**

The global efficiency (E) and the following nodal metrics were calculated for each graph and subsequently compared between groups: degree (d), local efficiency (E_loc_), and mean local efficiency (MeanE_loc_)_,_ was calculated using the Brain Connectivity Toolbox (Rubinov and Sporns, 2010). We calculated the integrated area under the global efficiency versus threshold curve (AUC_E_) to provide a summary measure independent of thresholding.

The global efficiency (E) quantifies the average inverse of the (characteristic) path length[^2^](#_ENREF_2), which itself quantifies the minimum number of edges (i.e. connections) that must be crossed to move between a given pair of nodes (i.e. ROIs;[^3^](#_ENREF_3)^,^ [^4^](#_ENREF_4)). Short characteristic path lengths are associated with greater global efficiency.

After confirming the data did not significantly deviate from the Normal distribution, the AUC_E_ was compared between groups using the univariate ANOVA with age as a covariate, gender as an explanatory variable and with False Discovery Rate (FDR) correction (q = 0.05) for two comparisons.

There were no significant differences in AUC_E_ between groups (BECTS group estimated marginal mean 0.11 standard deviation 0.003; siblings 0.11 ± 0.003; controls 0.11 ± 0.003; p = 0.77 uncorrected; Figure S2).

**Nodal metrics**

We used the Brain Connectivity Toolbox to calculate the local efficiency (E_loc_) for each node, which is akin to the global efficiency computed on node neighbourhoods, and similar to the clustering coefficient[^2^](#_ENREF_2). “Small-world” networks are characterised by both high global and local efficiency. The same software was used to calculate the degree (k), i.e. the number of edges connected to each node. For each node, we calculated the integrated area under the local efficiency (AUC_Eloc_) – and alternatively degree (AUC_d_) – versus threshold curves to provide summary measures independent of thresholding. We also calculated the integrated area under the mean local efficiency (AUC_MeanEloc_) versus threshold curves.

After confirming the data did not significantly deviate from the Normal distribution, the AUC_MeanEloc_ was compared between groups using the univariate ANOVA with age as a covariate, gender as an explanatory variable and with FDR correction (q = 0.05) for six comparisons. The AUC_Eloc_ and the AUC_d_ for each of 90 nodes was compared between groups using the univariate ANOVA as described above if applicable, or else using the non-parametric Kruskal Wallis test, with FDR correction (q = 0.05) for 90 comparisons.

There were no significant differences in AUC_MeanEloc_ between groups (BECTS group 0.14 ± 0.004; siblings group 0.14 ± 0.004; controls group 0.14 ± 0.004; p = 0.64 uncorrected; Figure S3). There were no significant differences in AUC_d_ or AUC_Eloc_ between groups for any node that remained significant after correction for multiple comparisons (p ≥ 0.006 and p ≥ 0.02, uncorrected, respectively).

**References**

1. Xia M, Wang J, He Y. BrainNet Viewer: a network visualization tool for human brain connectomics. PLoS One 2013; 8: e68910.

2. Latora V, Marchiori M. Efficient behavior of small-world networks. Phys Rev Lett 2001; 87: 198701.

3. Bullmore E, Sporns O. Complex brain networks: graph theoretical analysis of structural and functional systems. Nat Rev Neurosci 2009; 10: 186-98.

4. Watts DJ, Strogatz SH. Collective dynamics of 'small-world' networks. Nature 1998; 393: 440-2.
